# Asymmetric and Flexible Ag-MXene/ANFs Composite Papers for Electromagnetic Shielding and Thermal Management

**DOI:** 10.3390/nano13182608

**Published:** 2023-09-21

**Authors:** Xiaoai Ye, Xu Zhang, Xinsheng Zhou, Guigen Wang

**Affiliations:** 1 Guangdong Provincial Key Laboratory of Semiconductor Optoelectronic Materials and Intelligent Photonic Systems, School of Materials Science and Engineering, Harbin Institute of Technology (Shenzhen), Shenzhen 518055, China; 20b355007@stu.hit.edu.cn (X.Y.);; 2 National Key Laboratory of Science and Technology on Advanced Composites in Special Environments, Harbin Institute of Technology, Harbin 150080, China

**Keywords:** Ag-MXene/ANFs nanocomposite papers, asymmetric structure, EMI shielding, Joule heating performance

## Abstract

Lightweight, flexible, and electrically conductive thin films with high electromagnetic interference (EMI) shielding effectiveness and excellent thermal management capability are ideal for portable and flexible electronic devices. Herein, the asymmetric and multilayered structure Ag-MXene/ANFs composite papers (AMAGM) were fabricated based on Ag-MXene hybrids and aramid nanofibers (ANFs) via a self-reduction and alternating vacuum-assisted filtration process. The resultant AMAGM composite papers exhibit high electrical conductivity of 248,120 S m^−1^, excellent mechanical properties with tensile strength of 124.21 MPa and fracture strain of 4.98%, superior EMI shielding effectiveness (62 dB), ultra-high EMI SE/t (11,923 dB cm^2^ g^−1^) and outstanding EMI SE reliability as high as 96.1% even after 5000 cycles of bending deformation benefiting from the unique structure and the 3D network at a thickness of 34 μm. Asymmetric structures play an important role in regulating reflection and absorption of electromagnetic waves. In addition, the multifunctional nanocomposite papers reveal outstanding thermal management performances such as ultrafast thermal response, high heating temperatures at low operation voltage, and high heating stability. The results indicate that the AMAGM composite papers have excellent potential for high-integration electromagnetic shielding, wearable electronics, artificial intelligence, and high-performance heating devices.

## 1. Introduction

With the extensive use of personal electronic devices and the growing demand for increasingly powerful devices, electromagnetic interference (EMI) has risen to an unprecedented level [1,2]. EMI not only has a negative impact on people’s health, but also compromises performance of electronic devices by causing information leakage, false operations, or even complete failure [3,4,5,6,7]. Typical materials used for mitigating EM transmission including sheet metals, metal screens, and metal foams, cannot simultaneously integrate these intriguing characteristics due to opacity, high density, and inflexibility [8,9,10]. Recently, MXene are under the spotlight on account of their good electrical conductivity, light weight, extremely large specific surface areas, excellent hydrophilicity, and easy processability for efficient EMI shielding materials [11,12,13,14,15]. In addition, the Ti-F and Ti-OH bonds on the surface of the Ti_3_C_2_T_x_ nanosheets could be used as functional groups to reduce some metal ions, such as platinum ion, silver ions and gold ions, into corresponding metallic nanoparticles [16,17,18,19]. The reducing ability of Ti_3_C_2_T_x_ nanosheets not only presents an Ti_3_C_2_T_x_ nanosheets without an external reducing agent but also extends its application in various fields. Among the noble metals, silver has received tremendous attention due to its high electrical conductivity, good catalytic activity, and low cost. Guo et al. [6] fabricated PVA/MXene film featured with alternating multilayered structure through multilayered casting. The obtained multilayered film exhibited a maximum EMI SE of 44.4 dB at a low MXene loading of 19.5 wt%. Zhang et al. [20] reported an anisotropic porous multiwalled carbon nanotube/water-borne polyurethane composite, assembled via a facile freeze-drying method, which displays a high EMI shielding performance. Choi et al. [21] developed a lightweight, flexible, and conductive 3D porous Fe_3_O_4_@Ti_3_C_2_T_X_/GF/PDMS composite with an excellent conductivity up to 630 S m^−1^ and an outstanding average EMI SE of 80 dB in X-band. Although some exciting advances have been achieved, it remains an enormous challenge to manufacture EMI materials with ultrathin thickness, high flexibility, and excellent EMI shielding performance. Additionally, the spontaneous oxidation of MXene, particularly in aqueous dispersion form, is a key barrier which hinders its long-term usability during real-time applications [22,23].

Aramid nanofibers (ANFs), also called nanoscale Kevlar fibers, which can be extracted from poly (p-phenylene terephthalamide) (PPTA) fiber, are considered to be promising reinforcing phases and high-performance polymer substrates for various applications. ANFs exhibit good mechanical properties, impressive chemical and good thermal stability, excellent optical performance, and superior thermal resistance because of the high anisotropy ratio and strong interactions between PPTA chains such as hydrogen bonding, π–π stacking, and van der Waals forces. ANFs have rapidly gained significant attention in many areas such as battery separators, biological tissue, and electrical insulating materials [24,25,26,27,28]. Nonetheless, due to the insulating character of aramid nanofibers, it is imperative to improve the conductivity and EMI shielding performance of the composite paper prepared via direct mixing of MXene and cellulose nanofibrils. Therefore, to simultaneously improve the EMI shielding and mechanical performance, effective microstructural design and reasonable material selection and processing techniques should be considered for the fabrication of high-performance EMI shielding materials [29,30].

Herein, inspired by the millefeuille cake with multilayered and alternating aligned network structure between meringue sheets and cream components, we report asymmetric, ultra-flexible, and highly conductive Ag-MXene/ANFs composite papers through self-reduction followed by a facile alternating vacuum-assisted filtration technology for high-performance electromagnetic interference shielding and thermal management. Ag nanoparticles were easily introduced onto the surface of Ti_3_C_2_T_x_ nanosheets to form Ag-MXene hybrids via direct reduction of AgNO_3_, which is efficient to prevent MXene oxidation and improve the electrical conductivity. The sustainable and mechanically strong ANFs served as the polymer matrix to form extensive hydrogen-bonding interactions with Ag-MXene hybrids. Benefiting from the novel structure, the resultant nanocomposite paper exhibits not only outstanding mechanical properties (tensile strength, fracture strain, and toughness), but also excellent electrical conductivity. The composite films also provide excellent electromagnetic shielding capabilities and outstanding thermal management properties. The EMI SE reliability of bending deformation, conductive stability upon the repeated bending, long-term Joule heating are investigated in detail. We also discuss the asymmetric structures, which contribute to the value of SE_A_ and SE_R_ instead of the total EMI SE. Furthermore, EM field distribution simulation via COMSOL Multiphysics 6.1 was conducted to intuitively analyze the shielding mechanisms. This work provides a new strategy in manufacturing ultra-flexible and highly conductive composite paper for realizing outstanding EMI shielding performance and having promising potentials for next-generation wearable electronic devices in aerospace, military, and energy conversion.

## 2. Materials and Methods

### 2.1. Materials

Kevlar 49 thread was bought from DuPont (Delaware, DE, USA). Ti_3_AlC_2_ powders (400 mesh) were supplied by Foshan Xinxi Technology Co., Ltd. (Foshan, China). Chemicals including LiF, HCl, DMSO, KOH, AgNO_3_ were purchased from the Aladdin Reagent Co., Ltd. (Shanghai, China). Nylon membranes were provided by Xinya Purification Equipment Co., Ltd. (Shanghai, China).

### 2.2. Preparation of Aramid Nanofiber Dispersion

The stable ANFs dispersions were obtained according to the previously reported methods [31,32]. Typically, 2.0 g of Kevlar 49 thread and 3.0 g of KOH were added into 1000 mL of DMSO. The Kevlar/KOH/ DMSO suspension was magnetically stirred for 7 days at room temperature, obtaining a uniform and dark red ANFs dispersions. Then, a large amount of water was added to the above solution, and the KOH/DMSO system was filtered with large amounts of DI water to acquire ANFs precipitate. The obtained ANFs were washed with suction filtration, and redispersed well in deionized water to form a homogeneous ANF dispersion.

### 2.3. Preparation of Ti_3_C_2_T_x_ Nanosheets

Ti_3_C_2_T_x_ nanosheets were prepared through selectively etching Al [32]. In brief, LiF (3.2 g) was dissolved in HCl solution (40 mL, 9 M) in a Teflon beaker, assisted with magnetic stirring for 30 min to obtain the etching solution. The MAX powders (2 g) were added slowly to the Teflon beaker. The reaction was allowed to proceed for 48 h at 40 °C to obtain a clay-like Ti_3_C_2_T_x_ suspension, then the mixture was centrifuged using deionized water several times until the pH reached 6. After the sonication under N_2_ atmosphere for 60 min followed by centrifugation at 3500 rpm for 0.5 h, a homogeneous supernatant of Ti_3_C_2_T_x_ nanosheets was obtained and stored in a fridge for use.

### 2.4. Synthesis of Ag-MXene Hybrids

The Ag-MXene hybrids were prepared via direct reduction of AgNO_3_ [33]. Briefly, 200 mg of Ti_3_C_2_T_x_ MXene was uniformly dispersed in 100 mL water via ultrasound for 30 min. Then, the AgNO_3_ solution (50 mg) was injected into the MXene solution drop by drop and placed under continued stirring for another 30 min. Finally, the suspension was centrifuged and washed with water and ethanol, respectively. AM-10, AM-15 composites were also prepared under the same conditions except for the mass of AgNO_3_ (100 mg, and 150 mg).

### 2.5. Preparation of Ag-MXene/ANF Composite Paper with Asymmetric and Multilayered Structure (AMAGM)

The AMAGM composite papers were fabricated via the alternating vacuum-assisted filtration method. First, the ANF dispersion (10 mg) was vacuum filtrated onto a porous nylon membrane until a steady ANF hydrogel was obtained. Subsequently the desired AM-5 (10 mg) dispersion was sedimented on the top of the ANF hydrogel using the identical method. Then, an ANF (10 mg) layer was deposited on the top of AM-5 in the same way. After that, AM-10 (10 mg), ANFs (10 mg), AM-15 (10 mg), and ANFs (10 mg) were successively added to deposit on their former layer. The composite papers were completely dried in vacuum oven at 60 °C for 12 h. Other composite papers with gradient and multilayered structure can be prepared through a similar process. For comparison, the pure ANF papers and a randomly mixed Ag-Ti_3_C_2_T_x_ MXene (30 mg)/ANFs (8 mg) composite paper (abbreviated as AMA mixture) were also manufactured via a similar vacuum-assisted filtration.

### 2.6. Characterization

The crystalline characterization information of all prepared samples was identified by X-ray diffractometer (XRD, Rigaku D/MAX-2500/PC, Tokyo, Japan) with Cu Kα radiation of λ = 0.15418 nm. FT-IR absorption spectra were collected using FTIR spectrometer (Nicolet iS10 FT-IR system, Thermo Fisher Scientific, Waltham, MA, USA). The surface morphologies and microstructures of AMAGM composite films were observed with AFM (Bruker Dimension Icon, Saarbrücken, Germany), SEM (Hitachi S-4700, 20 kV, Tokyo, Japan), and HADDF-STEM (G2 F20, Tecnai, Hillsboro, OR, USA). Elemental mapping analyses were carried out by EDS. The surface chemical composition was characterized by XPS (K-Alpha+, Thermo-Fisher, San Diego, CA, USA). The mechanical properties of AMAGM composite films were tested using a universal testing machine (Japan Instrumentation System Co., Ltd., Tokyo, Japan with rectangular strips of 5 × 30 mm with loading strain rate of 0.2 mm/min) with a force sensor of 500 N (JLC-M500N, Tokyo, Japan). Electrical conductivity was measured with the standard four-point using Loresta-GP meter (MCP-T610, Mitsubishi Chemical, Tokyo, Japan Japan). The surface wettability was analyzed by using an OCA20 optical contact angle measurement apparatus. The thermal stability was evaluated by using a thermogravimetric analyzer (TGA, Netzsch, STA449F5, 10 °C min^−1^, Bavaria, Germany).

### 2.7. EMI SE Performance Tests

A 2-port vector network analyzer (Rohde–Schwarz ZNA43, Munich, Germany) was employed to analyze the EMI SE of the nanocomposite papers (22.86 × 10.16 mm^2^) in X-band. The scattering parameters (S_11_, S_12_, S_22_, S_21_) output by the vector network analyzer were applied to analyze the shielding effectiveness. Each sample was tested at least three times, and the final result was taken as the average value. The total EMI SE (SE_T_) contributions from reflection (SE_R_), absorption (SE_A_), and multiple internal reflections (SE_M_) were calculated as follows:

(1) $$R={\left|S_{11}\right|}^{2}={\left|S_{22}\right|}^{2},\;T={\left|S_{12}\right|}^{2}={\left|S_{21}\right|}^{2}$$

(2) A=1−R−T

(3) $$SE_{R}=-10\log\left(1-R\right)$$

(4) $$SE_{A}=-10\log\left(\frac{T}{1-R}\right)$$

In addition, to further evaluate the absolute shielding performance of the films equitably, surface density and the thickness of the materials were also taken into account:

(5) $$SSE=EMI\frac{SE}{density}=dB\;cm^{3}g^{-1}$$

(6) $$SSE∕t=SSE|thickness=dB\;cm^{2}g^{-1}$$

Specifically, EMI shielding efficiency (%), meaning the ability to block waves in terms of percentage, can be obtained using this equation:

(7) $$\mathrm{Shielding}\;\mathrm{efficiency}\;(\%)=100-\left(1∕100^{SE∕10}\right)\times 100$$

## 3. Results and Discussion

### 3.1. Microstructures and Characterization

**Figure 1:**
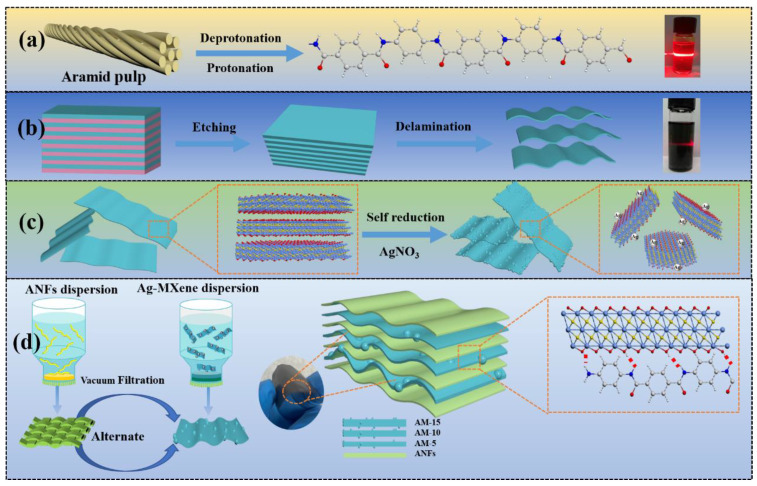
Schematic diagram of the fabrication procedure. The preparation of (**a**) ANFs and (**b**) Ti_3_C_2_T_x_ MXene; (**c**) process for in situ reduction of AgNPs on Ti_3_C_2_T_x_ MXene; (**d**) schematic illustrating the fabrication of AMAGM composite paper.

The homogeneous ANFs dispersion were prepared by the deprotonation of macroscopic aramid fabrics as exhibited in Figure 1a. The micro-sized ANFs were transformed from the macroscale Kevlar fibers by abstracting the mobile protons from amide groups, weakening the hydrogen-bonding interactions between polymer chains and strengthening the electrostatic repulsion under the KOH/DMSO system. The as-prepared ANFs nanofibers display a length of several micrometers with a high-aspect-ratio and a radial size of 11 nm (Figure S1a,b). The preparation process of 2D Ti_3_C_2_T_x_ nanosheets is illustrated in Figure 1b. The (002) characteristic diffraction peak of Ti_3_C_2_T_x_ MXene shifted to a smaller angle than Ti_3_AlC_2_ powder (9.70°) (Figure S2a,b). The as-obtained Ti_3_C_2_T_x_ MXene exhibits a typical 2D lamellar structure with a lateral size of approximately 2 µm and high dispersibility due to electrostatic repulsion caused by negatively charged terminal groups such as -OH, -F, and = O. The thickness of individual MXene nanoflakes is about ~1.0 nm, suggesting the Ti_3_C_2_T_x_ nanosheets are mostly single-layer (Figure S2c–f). The fabrication process for Ag-MXene hybrids is depicted in Figure 1c. The Ti_3_C_2_T_x_ nanosheets can be used as reducing agent to synthesize Ag nanoparticles on the surface layers due to the negatively charged terminal groups (Ti-OH and Ti-F) [34]. The chemical compositions and elemental components were characterized via XPS (Figure S3a). The characteristic peak of silver element appears in the XPS wide-scan spectra of the AM hybrid film. The obvious peak, ranging from 368–374.5 eV, demonstrates the presence of Ag nanoparticles in the sample. To further analyze the role of the content of silver, AM-5, AM-10, and AM-15 samples were conducted for the X-ray diffraction (Figure S3b). The AM-5 XRD pattern presents not only a strong (002) diffraction peak of 7.5°, assigned to Ti_3_C_2_T_x_ nanosheets, but also a series of weak peaks belonging to the (1 1 1), (2 0 0), (2 2 0), and (3 1 1) planes of face-centered cubic Ag single crystal [35,36]. With the increase in AgNO_3_, the peaks of Ag become sharper and stronger, while the (002) peak intensity of the Ti_3_C_2_T_x_ nanosheets becomes weaker, indicating the partial oxidation of the Ti_3_C_2_T_x_ nanosheets during the reduction reaction process of silver nanoparticles [37]. TEM and SEM images were used to characterize the morphology and microstructure of the AM-5 nanocomposites (Figure S3c–f). Some non-uniform-sized nanoparticles distributed on the surface of Ti_3_C_2_T_x_ nanosheets are silver particles, and it was further verified via energy dispersive spectrometer, meaning the successful introduction of Ag nanoparticles onto Ti_3_C_2_T_x_ nanosheets.

**Figure 2:**
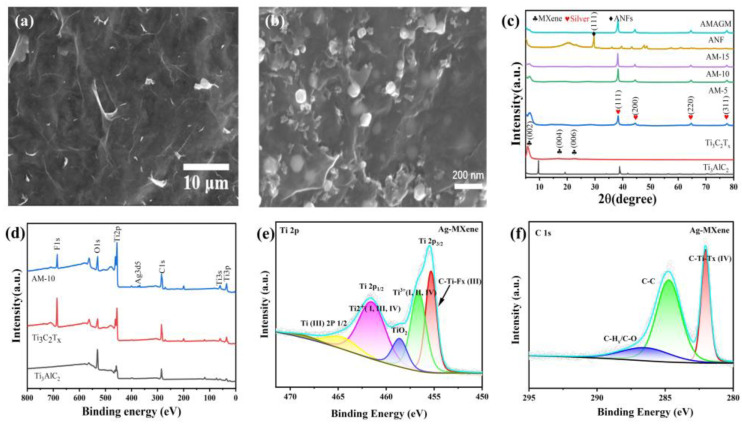
Morphology and structure characterization. (**a**) SEM images of Ti_3_C_2_T_x_ nanosheet paper. (**b**) SEM images of Ag-MXene composite paper. (**c**) XRD patterns of MXene, ANFs and AMAGM nanocomposite papers. (**d**) XPS surveys of delaminated Ti_3_C_2_T_x_ nanosheets and Ag-MXene nanocomposites. (**e**) High-resolution XPS spectra of Ti 2p for Ag-MXene hybrids. (**f**) High-resolution spectra of C 1s for Ag-MXene.

Figure 1d schematically illustrates the fabrication process of AMAGM composite papers via vacuum-assisted alternating filtration of ANFs and Ag-Ti_3_C_2_T_x_ MXene dispersion. It is worth noting that the outer surfaces on two sides of the multilayered film are ANF layers. The composite paper can be easily achieved through this method without any valuable equipment. The dense ANF layer can prevent the entry of oxygen to some extent, tightly wrap the AM layer, and prevent the oxidation of silver under high humidity conditions. In this bargain, a construction strategy is beneficial for improving both mechanical properties and EMI shielding performance. In the atomic model, MXene nanosheets are tightly interacted with hydrogen bonding by the ANFs to form the “stiffeners−interlocks” structure illustrated in Figure 2h [38]. Nanocomposite paper has sufficient flexibility and mechanical properties, which is of great significance for designing high-performance EMI-shielding materials that are resistant to mechanical deformation. The as-obtained composite paper is ultrathin with a smooth surface, and can be folded into a complicated model without cracking, showcasing the outstanding flexibility (Figure S4a–c). Furthermore, the thickness is only 34 µm, which is significant in practical applications, especially next-generation portable equipment and wearable devices.

SEM imaging was carried out to further observe the morphology and microstructure. The top-view and cross-sectional SEM images of the Ti_3_C_2_T_x_ nanosheets paper and Ag-MXene hybrid are displayed (Figure 2a,b and Figure S5a,b). It is obvious that the pure Ti_3_C_2_T_x_ nanosheets paper possess some wrinkles and the surface is coarse, while the cross-section presents multi-layered, stacked architecture. As for the Ag-MXene hybrid, some urchin-like and non-uniform nanoparticles are embedded on the surface. The amount and particle size of silver nanoparticles on the surface of MXene are related to the silver nitrate contents. EDX results testify that Ag nanoparticles have been attached onto Ti_3_C_2_ nanosheets. It is worth noting that the stacked-layer morphology of MXene does not change obviously during the self-reduction process. Figure 2c exhibits the XRD patterns of the pure MXene paper, Ag-MXene hybrid, and AMAGM composite paper. The shift of the characteristic peak (002) from 9.6° to 7.9° and the appearance of another two prominent diffraction peaks corresponding to the (004) and (110) demonstrate the successful exfoliation of Ti_3_C_2_T_x_ sheets. The appearance of four pronounced diffraction peaks indexed to the (111), (200), (220), and (311) planes of face-centered cubic single silver crystals is in accordance with previous reports [32,39,40]. These results confirm the successful synthesis of Ag-MXene hybrids. The retained characteristic peaks (002) in composite paper demonstrate the well-preserved laminated structure of Ti_3_C_2_T_x_ nanosheets. This proves the good combination between ANFs and Ag-MXene hybrids because only diffraction peaks resulting from (111) and (200) planes of MXene and the (110) plane of ANFs can be examined. The Fourier transform infrared spectra and XPS patterns were performed to analyze the valence bond and the chemical compositions (Figure 2f). As shown in the FT-IR spectrum of Ti_3_C_2_T_x_ nanosheets, a broad and strong absorption band near 2158 and 2020 cm^−1^ were observed, which is ascribed to the stretching vibration of the hydroxyl (-OH) and C−O. As for the FTIR spectrum of ANFs, the characteristic peaks located at 3335, 1540 and 1645 cm^−1^ correspond to the stretching vibration of N−H, deformation of N−H, and stretching vibration of C=O, respectively. For the AMAGM composite paper, there are some subtle differences in the film. The stretching vibration of the hydroxyl (-OH) is red-shifted to 3436 cm^−1^, while the characteristic peak of C=O shifts from 3440 to 3432 cm^−1^, suggesting the chemical environment has been changed, which might be attributed to the formation of hydrogen bonds among ANFs and Ag-MXene hybrids (Figure S5c). Besides, XPS analysis was performed to examine the surface chemical bonding and electrostatic interaction of Ag-MXene nanocomposite and Ti_3_C_2_T_x_ nanosheets. As displayed in Figure 2d–f and Figure S5d–f, the XPS wide-scan spectra of the Ag-MXene hybrids demonstrates the existence of Ag elemental except Ti, C, O, and F elemental. The characteristic peaks at 368.4 eV and 374.4 eV corresponding to Ag 3d_5/2_ and Ag 3d_3/2_ peaks, respectively, which are in accordance with the existing literature, suggest the successful reduction of Ag (0). For the high-resolution spectra of Ti 2p observed from the pure Ti_3_C_2_T_x_ nanosheets via peak splitting, the characteristic peaks at 455.50, 456.16, 457.23, 461.97, and 461.99 eV belong to Ti^2+^(I, and II), Ti^2+^(I, and III), Ti^3+^ (I, and II), C-Ti-F_x_ (III), C-Ti (III) 2P_1/2_, separately. After the self-reduction process, the low-valence Ti species was replaced by an intermediate Ti (IV) species due to the emergence of stable TiO_2_ formation. Additionally, there is a new peak around 459.41 eV, assigned to TiO_2_ for Ag-MXene hybrids, confirming the strong reductive activity on the low-valence Ti species and the existence of Ag nanoparticles. As shown in the deconvoluted C1s spectrum, the distinct peak located at 282.52, 284.60, and 287.21 eV are assigned to C-Ti-T_x_, C-C, and CH_x_/C-O. After treatment with AgNO_3_, the split peaks of Ti 2p are shifted to binding energy positions of 284.59, 281.98, 287.73 eV, ascribed to C-C, CH_x_/C-O, and C-Ti-T_x_ (IV), which may be due to the slight oxidation of Ti_3_C_2_T_x_ nanosheets formed via the reduction of silver ions [32].

### 3.2. Electrical and Mechanical Properties

**Figure 3:**
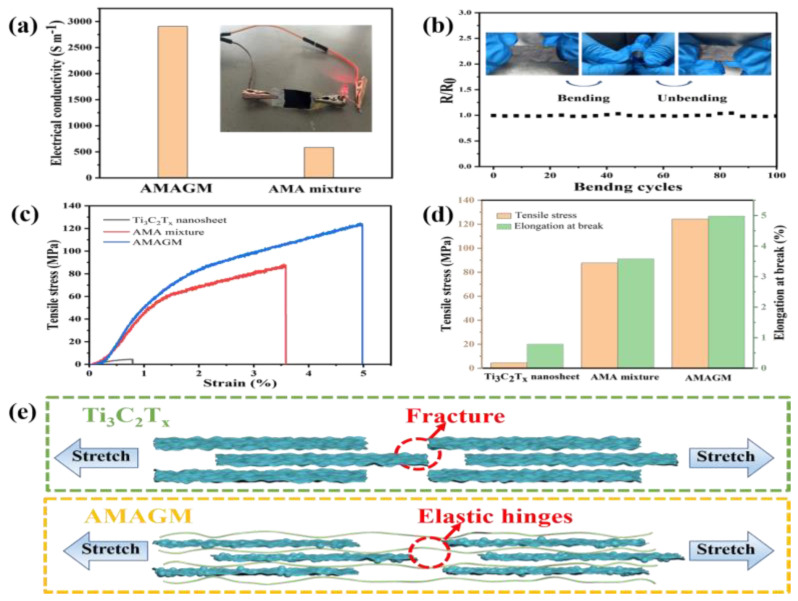
Property and structure characterizations of flexible composites. (**a**) Light an LED bulb. (**b**) Electrical resistance variation of AMAGM composite paper with bending test. (**c**) Tensile stress–strain curves of the pure Ti_3_C_2_T_x_ nanosheet, AMAGM composite paper, and AMA mixture. (**d**) Pure Ti_3_C_2_T_x_ nanosheet, AMAGM composite paper, and AMA mixture, respectively. (**e**) Schematic illustrations of the fracture mechanism of pristine Ti_3_C_2_T_x_ nanosheet and AMAGM composite paper.

Electrical conductivity is of great significance for electromagnetic interference shielding materials. As displayed in Figure 3a, we lit an LED bulb successfully via the AMAGM nanocomposite paper at 1.5 V external voltage, suggesting good electrical conductivity. We also found that the conductivity of the AMAGM composite paper is 5-fold higher than that of AMA mixture film. This increasing electrical conductivity and the asymmetric structure endows the AMAGM composite paper with superior EMI-shielding ability. The resistance variation of is also explored upon repeated bending and stretching tests. It displays a stable relative resistance R/R_0_ even after 100 bending cycles at different curvatures (Figure 3b, R/R_0_, between 0.996 and 1.018). The high flexibility and robust layered structure can effectively prevent damage to the conductive network to some extent, which significantly improves the practical potential in wearable or portable electronic devices. Adequate flexibility and mechanical properties are of great significance for the design of EMI-shielding materials to endure mechanical deformation, especially in the field of flexible electronic devices. The tensile stress−strain curves are displayed in Figure 3c,d. The pure Ti_3_C_2_T_x_ nanosheets exhibits poor mechanical properties with an extremely low tensile strength of 4.421 ± 0.47 MPa and a fracture strain of 0.79 ± 0.18% due to the weak interaction, which severely restricts their applications in harsh environments. Compared to pure Ti_3_C_2_T_x_ nanosheets, the randomly assembled AMA mixture composite paper shows excellent mechanical performance with a tensile strength of 87.76 ± 4.4 MPa and a fracture strain of 3.61 ± 0.2%, which are 19.8 and 4.5 times higher than those of pure Ti_3_C_2_T_x_ nanosheets, respectively. Generally speaking, ANFs are usually considered an ideal reinforcement because of their rigidity and interpenetrating network. Evidently, the successful incorporation of ANFs has a significant improvement on the tensile stress and strain of the AMA mixture. In particular, the AMAGM composite paper presents a better mechanical performance with a tensile strength of 124.21 ± 11.3 MPa and a fracture strain of 4.98 ± 1.4% compared with the AMA mixture. This is probably ascribed to forming an interconnective network and extensive hydrogen-bonding interactions, which can effectively distribute stress and obtain high mechanical properties. The hydrogen bonding interaction may be formed between the Ag-MXene conductive layer and the ANFs enhancement layer, thus avoiding the cracking of Ag-MXene conductive layer due to external force.

To explore the enhancement of ANFs on the mechanical properties of the AMAGM composite paper, the tensile failure processes of the pure Ti_3_C_2_T_x_ nanosheet film and AMAGM composite paper are proposed to elucidate the mechanism in Figure 3e. When subjected to tensile load, the pure Ti_3_C_2_T_x_ nanosheets can be inclined to slide over each other and will be stretched into an elastic limit state. Then, the Ti_3_C_2_T_x_ nanosheets will suffer partial interlayer gliding and rearrangement till the whole film eventually turns into a tightening state. Finally, the film achieves a complete fracture at a strain of 0.8%. Even the Ag-MXene hybrids are completely separated during the further stretching process in AMAGM composite paper, the ANFs as the elastic hinges can still connect the separated nanosheets until the hydrogen bonds are slowly destroyed. The ANF chains can be stretched during the elastic deformation process till fracture occurs, which results in an ultimate crack. This asymmetric structure of the composite paper contributes to greatly enhancing the tensile strength and fracture strain of the AMAGM composite film. This long-term stability of electrical conductivity makes the composite film is promising for commercial application in extreme environments.

**Figure 4:**
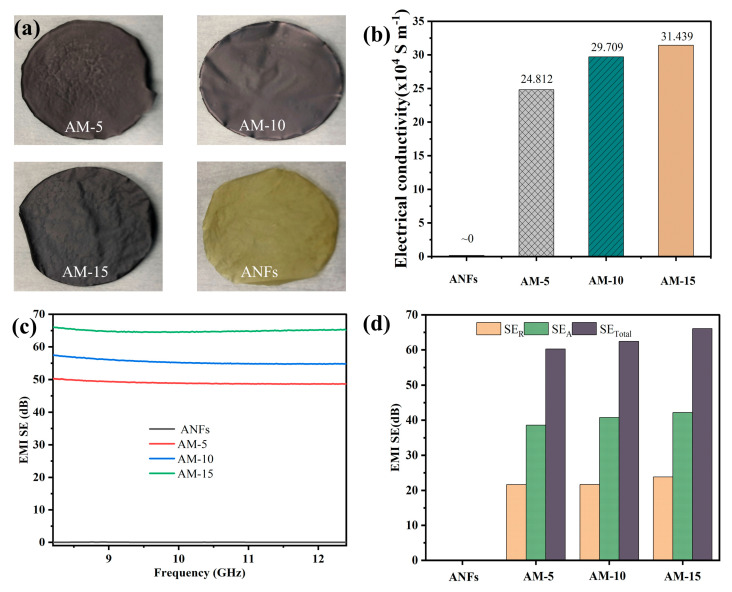
Property and EMI shielding efficiency of Ag-hybrids. (**a**) Digital images of Ag-MXene hybrids and ANF film. (**b**) Electrical conductivity; (**c**) EMI SE; and (**d**) SE_T_, SE_A_, and SE_R_ of AM-5, AM-10, AM-15, and single-layered ANFs.

The digital images of single-layered ANFs, AM-10 and AM-15, respectively, which constitute the AMAGM composite film, are exhibited in Figure 4a. Figure 4b shows the electrical conductivity for each component, which is crucial to the conductive materials for Joule heating and EMI-shielding performance. This indicates that single-layered ANFs seem to be an insulator and possesses no electrical conductivity, while the AM-5 hybrid exhibits an electrical conductivity of 248,120 S m^−1^, which is far higher than requirement for the commercial standard (1 S m^−1^). With increasing silver-loading contents, the acquired AM-15 achieves an ultrahigh electrical conductivity of 314,390 S m^−1^, demonstrating the formation of efficient conductive networks. As expected, the highly conductive Ag-MXene hybrids show outstanding prospects for EMI shielding applications in the X-band at room temperature (Figure 4c). The pure ANFs are almost transparent to the incident electromagnetic waves due to insulation characteristics, and their EMI SE can be neglected. The total EMI SE of Ag-MXene hybrids gradually improves with increasing silver-loading contents because of the lower sheet resistance. For AM-15 film, it reveals an excellent EMI SE of 66 dB at the frequency of 8.2 GHz, and the contribution of SE_R_ is only 34.3% (Figure 4d).

### 3.3. Electromagnetic Shielding Performances

**Figure 5:**
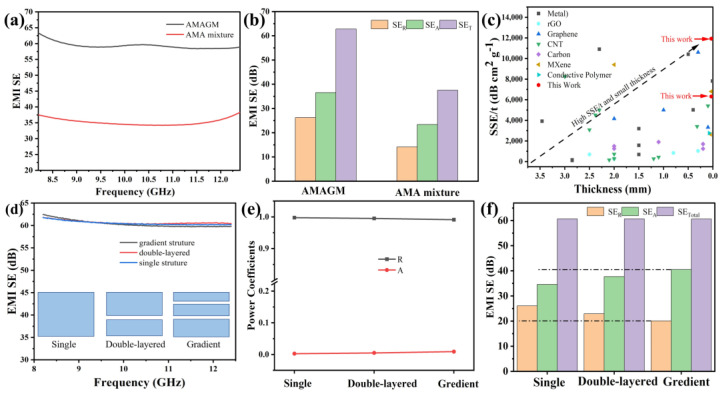
EMI-shielding performance of AMAGM composites. (**a**) EMI SE of composite paper with different structures in the X-band. (**b**) Comparison of SE_T_, SE_A_, and SE_R_. (**c**) Comparison of the specific EMI shielding effectiveness as a function of thickness. (**d**) EMI SE with different structures. (**e**) R and A of composite paper with different structures in 8.2 GHz. (**f**) Comparison of SE_T_, SE_A_, and SE_R_ of composite paper with different sandwich structures.

Figure 5a provides the total EMI shielding effectiveness (SE_T_) of AMA mixture film and AMAGM composite paper in the X-band. The EMI SE of AMA mixture film is 37.5 dB (larger than the industrial standard of 20 dB), which meets the requirements for practical applications of commercial electromagnetic shielding materials. Additionally, the AMAGM composite paper presents a strong EMI-shielding ability to obstruct electromagnetic waves with an average EMI SE about 62 dB, which indicates only the transmission of 0.0004% incidental wave. It has been observed that SEA contribute to SE_T_ mostly, suggesting that the EMI-shielding performances are mainly determined by the absorption of electromagnetic waves [41]. The mechanical deformation was performed systematically to analyze the long-term stability of the EMI shielding for the AMAGM composite paper. As can be seen, even after 5000 cycles of bending deformation, the AMAGM composite paper shows a very slight variation (~3.9% reduction, Figure S6), showcasing excellent EMI shielding stability, which is ascribed to introduction protective coating of ANFs on the top and bottom sides. In general, the AMAGM composite paper possesses outstanding EMI-shielding reliability and huge potential in harsh environments. The thickness, density, and the EMI SE are three important factors for EMI materials. Up to now, carbon-based, metal-based, and other composites are the most widely used shielding materials. Although some of the literature has reported ultra-high SSE/t EMI-shielding materials, such as Ag@C core–shell sponges (SSE/t of 61,169 dB cm^2^ g^−1^) [42], GO/Ag-7L (SSE/t of 77,500 dB cm^2^ g^−1^) [43], and CNT/PU@PU sponges (SSE/t of 22,316 dB cm^2^ g^−1^) [44], few studies have reported polymeric EMI-shielding composites that possess flexibility, ultra-thin thickness, and excellent EMI-shielding performance at the same time. In this work, the as-obtained AMAGM composite film exhibits both ultra-thin thickness (34 µm) and high SSE/t (11,923 dB cm^2^ g^−1^), which is superior to other shielding materials (seen in Figure 5c and Table S1).

To explore the effect of asymmetric and sandwich structures on the EMI SE of the AMAGM composite film, some single- or double-layered AM composite paper with various Ag-MXene contents in each layer has also been prepared via a similar vacuum-assisted filtration technology. In particular, the single structure refers to a homogeneous layer composed of Ag-MXene hybrids (10 mg AM-5 + 10 mg AM-15 + 10 mg AM-15), and the double-layered structure consists of an upper layer (10 mg AM-5 + 10 mg AM-15) and lower surface (10 mg AM-10). As displayed in Figure 5d, there is almost no significant difference in electromagnetic shielding performance among the samples throughout the X-band. We also calculated the reflection coefficient (R) and absorption coefficient (A) individually to analyze the underlying EMI shielding mechanism. Obviously, the R and A of each sample with different structure are quite different, and R is always higher than A. The R values show a correlation of single- > double-layered > gradient structure, while the A values present a rapid upward trend, which is also revealed by the representation corresponding to the increment in SE_A_ displayed in Figure 5f. It can be deduced that the asymmetric structures significantly affect the value of SE_A_ and SE_R_ rather than their total EMI SE [45].

### 3.4. Shielding Mechanism and Application

**Figure 6:**
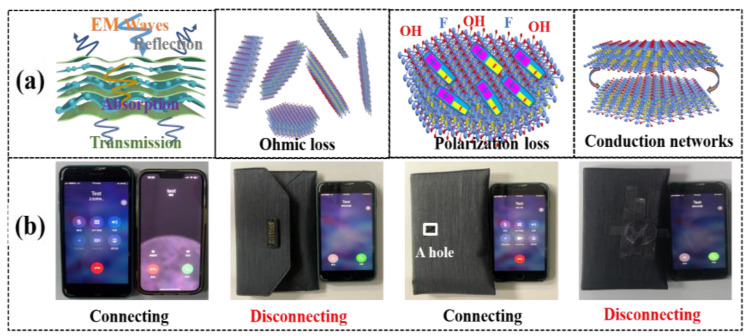
EMI-shielding mechanism and application. (**a**) EMI shielding mechanism of the AMAGM nanocomposite paper. (**b**) The realistic application of the AMAGM composite paper for EMI shielding.

Figure 6a illustrates the proposed mechanism of the AMAGM composite paper for EMI shielding. As the initial incident electromagnetic microwave struck the outside ANF layer, only a little electromagnetic microwave reflected; most of the electromagnetic microwave was transmitted through the ANFs layer. When the most remaining electromagnetic waves make contact with the AM layer, some incident waves are immediately reflected due to the high electrical conductivity and their impedance mismatch of the continuous AM layer at the interface between air and AM layer [46]. During this period, a part of the electromagnetic waves is lost because of the silver nanoparticles’ natural resonance on the MXene upper surface. After that, when the electromagnetic waves pass through the AM layer, the remaining electromagnetic waves would undergo polarization loss and ohmic losses as it interacts with the terminating functional groups and high-density electron carriers, thus leading to drastic attenuation of the energy of electromagnetic waves. Furthermore, the overall parallel MXene sheets enables the AM layer to behave as a multilevel shield, which can make the electromagnetic waves suffer massive ohmic losses and be reflected between the adjacent MXene nanosheets, the electromagnetic waves would be further dissipation due to the slit-shaped micropores and the high conductivity of AM layer [13]. This is an important way to attenuate the incident waves. The entered electromagnetic waves were trapped and converted into heat and other forms of energy in the form of microcurrent, significantly enhancing the EMI-shielding efficiency. After that, as the surviving incident electromagnetic waves enter the layered structure of the AMAGM paper, they were further attenuated progressively by the multiple reflection. The repeated reflection and scattering can greatly attenuate or eliminate the internal electromagnetic waves and further achieve an excellent EMI-shielding performance [47]. Besides, the service time of the AMAGM composite papers can be extended because of the protection of the layered structure from oxidation. Therefore, we surmise that the excellent EMI shielding performance of the AMAGM paper was principally triggered by the improved absorption and multiple reflection [38,48].

Furthermore, a practical application was performed to demonstrate the EMI shielding ability of the AMAGM composite paper. A mobile phone can be freely connected with another one, as illustrated in Figure 6b. When one phone is placed in the shielding bag, the connection between the two phones is cut off because of the blocked signal resulting from the shielding bag. When the shielding bag is broken with a square hole, the phones can be smoothly connected with each other owing to the electromagnetic wave leaked through the hole. After the hole is covered by the AMAGM composite paper, indicating the electromagnetic wave being shielded again, the phones disconnect [49,50].

### 3.5. Simulation of EMI Shielding Properties

**Figure 7:**
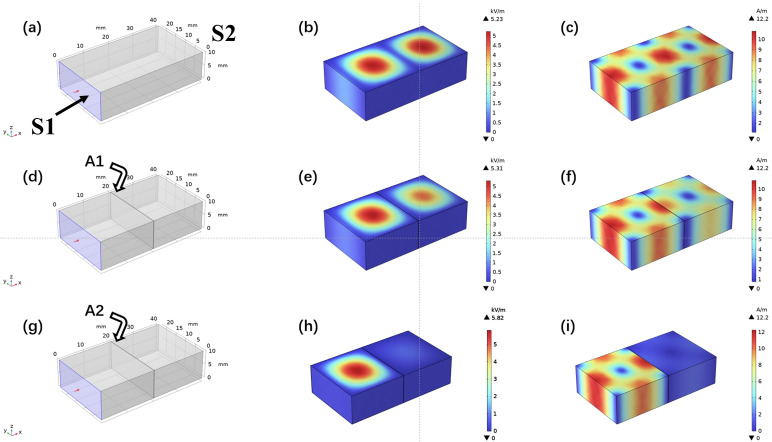
EMI shielding performance simulation of different samples. (**a**,**d**,**g**) The simulation models for shielding performance. (**b**,**e**,**h**) The electric field simulation for different models. (**c**,**f**,**i**) The magnetic field simulation for different models.

COMSOL Multiphysics was used to simulate the EMI-shielding properties of the samples. To further explain and describe the shielding mechanism, the simulation model was utilized to analyze the electric field and magnetic field distributions in COMSOL Multiphysics 6.1 (Figure 7). The size of the model was set as 22.86 × 10.16 mm^2^, similar to the waveguide. The model approximates the walls with ideal conductors, represented by the boundary condition n × E = 0, allowing EM waves to propagate only within the waveguide. The left boundary of the model was set as the excitation source, and numerical ports were used for calculations. The excitation frequency was set to 10 GHz. Finally, the relevant material parameters such as complex permittivity and complex permeability were inputted into the model [51].

It shows the distribution of electric and magnetic fields within the waveguide as EM waves enter it (Figure 7a–c); the distribution of electric and magnetic fields is strong and almost identical, indicating that the waves propagate smoothly through the waveguide model without loss [52]. Figure 7d–f displays the insertion of shielding material ANFs into the waveguide. The intensity of the EM field and magnetic field show little change, suggesting that ANFs possesses the inferior attenuation ability for EM waves and weak EMI shielding properties, which is consistent with the experimental results. On the contrary, as can be seen in Figure 7g–i, the intensity of the electric field enhances significantly, demonstrating the strong interactions between AMAGM and the EM waves after the insertion of shielding material AMAGM into the waveguide. After passing through AMAGM, the electric and magnetic fields rapidly attenuate, indicating that AMAGM can effectively shield EM waves.

### 3.6. Thermal Management Performances

**Figure 8:**
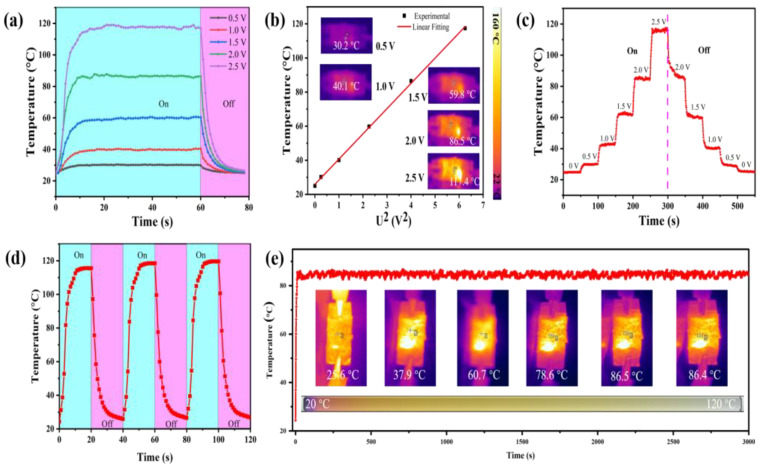
Rapid electrothermal conversion of multilayered AMAGM composites. (**a**) Time-dependent surface temperatures. (**b**) Experimental data and linear fitting of saturation temperature versus U^2^. (**c**) Tailored surface temperatures upon gradient-changed voltages. (**d**) Cyclic test. (**e**) Long-term time−temperature curve at a constant voltage of 2.0 V.

Excluding the charming features mentioned above, the flexible AMAGM nanocomposite papers can be used as high-performance electric heaters based on electrothermal conversion. Figure 8a presents the time-dependent surface temperature curves of prepared AMAGM composite paper (2.5 × 1.5 cm^2^) under different DC voltages from 0.5 to 2.5 V. According to Joule’s equation: $Q=\frac{U^{2}}{R}t$. It is clear that the heat engendered is proportional to the applied voltage [53]. The electric current passing through the AMAGM nanocomposite paper becomes higher as soon as the voltage applied on its two ends increases, generating more Joule heat because of the inelastic collision between accelerated electron and phonon [54,55]. It takes less than 10 s for the flexible AMAGM nanocomposite papers to rapidly reach the saturated temperature of 30, 47, 73, 101, 138 °C under low voltages of 0.5, 1.0, 1.5, 2.0, and 2.5 V, respectively, benefiting from the construction of effective multilayered structures and three-dimensional Ag-MXene hybrid conductive networks. As for the flexible and multilayered Ag-MXene/ANFs composite paper, there is an excellent linear relationship between the steady-state temperature and the square of the applied voltage, as demonstrated in Figure 8b, which conforms to Joule’s law, proving a controllable Joule heating performance by just tuning the supplied voltage. Figure 8c reveals that the surface temperatures of the nanocomposite papers can be rapidly switched by applying different gradients of DC voltage in real time, either from 0 to 2.5 V or from 2.5 to 0 V, due to the ability to respond quickly to the change in continuous voltages. Furthermore, it means that the electric heaters can be powered by a portable battery, owing to the low driving voltage [56]. The time-dependent temperature of the flexible AMAGM nanocomposite papers under a constant voltage of 2 V for 3000 s was recorded via infrared thermal imaging to assess the heating recyclability and stability. This also demonstrates that the film possesses an excellent cyclic on−off thermal response at the applied voltage of 2.5 V (Figure 8d). As exhibited in Figure 8e, the nanocomposite papers present a stable temperature with negligible resistance changes after reaching the saturated value (around 101 °C), demonstrating their superior durability and reliability. By virtue of low driving voltage, fast thermal response, high Joule heating temperature, and long-term stability, the flexible and multilayered Ag-MXene/ANF composite paper is conductive to the thermal management applications [57,58].

## 4. Conclusions

In summary, Ag-MXene hybrids composed of metal nanoparticle-decorated oxidized MXene were successfully fabricated via a self-reduction process. The loading content of silver nitrate treatment plays a vital role in the conductivity. We have manufactured a flexible and conductive Ag-MXene/ANFs composite paper with an asymmetric structure via a facile, vacuum-assisted alternating filtration technique. The prepared AMAGM composite paper demonstrates excellent mechanical properties with a tensile strength of 124.21 MPa and a fracture strain of 4.98%, thanks to extensive hydrogen-bonding interactions and the tight intertwining ANF substrate. The resultant AMAGM composite paper exhibits a superior EMI SE of 62 dB, high EMI SE/t of 11,923 dB cm^2^ g^−1^, and high EMI SE retention (96.1%) even after 5000 cycles of bending deformation, which is better than the randomly mixed AMA mixture (37.5 dB). Meanwhile, the multifunctional composite paper displays outstanding thermal management performance such as wide Joule heating temperature (25–128 °C), a sensitive response (<10 s), safe supplied voltages (0.5–2.5 V), and long-term stability (3000 s). Thus, these multifunctional AMAGM composite papers are have promising applications in high-integration electromagnetic shielding, wearable electronics, artificial intelligence, and high-performance heating devices.

## Data Availability

The data presented in this study are available on request from the corresponding author.

## Supplementary Materials

The following supporting information can be downloaded at: https://www.mdpi.com/article/10.3390/nano13182608/s1. References [59,60,61,62,63,64,65,66,67,68,69,70,71,72,73,74,75,76,77,78,79,80,81,82,83,84,85,86,87,88,89,90,91,92,93,94] are cited in the supplementary materials.

## References

[1] Yang S., Yang P., Ren C., Zhao X., Zhang J. (2022). Millefeuille-inspired highly conducting polymer nanocomposites based on controllable layer-by-layer assembly strategy for durable and stable electromagnetic interference shielding. J. Colloid Interface Sci..

[2] De A., Bera R., Paria S., Karan S.K., Das A.K., Maitra A., Si S.K., Halder L., Ojha S., Khatua B.B. (2020). Nanostructured cigarette wrapper encapsulated PDMS-RGO sandwiched composite for high performance EMI shielding applications. Polym. Eng. Sci..

[3] Yao B., Hong W., Chen T., Han Z., Xu X., Hu R., Hao J., Li C., Li H., Perini S.E. (2020). Highly Stretchable Polymer Composite with Strain-Enhanced Electromagnetic Interference Shielding Effectiveness. Adv. Mater..

[4] Yang X., Wang Q., Zhu K., Ye K., Wang G., Cao D., Yan J. (2021). 3D Porous Oxidation-Resistant MXene/Graphene Architectures Induced by In Situ Zinc Template toward High-Performance Supercapacitors. Adv. Funct. Mater..

[5] Zeng Z., Jiang F., Yue Y., Han D., Lin L., Zhao S., Zhao Y.-B., Pan Z., Li C., Nyström G. (2020). Flexible and Ultrathin Waterproof Cellular Membranes Based on High-Conjunction Metal-Wrapped Polymer Nanofibers for Electromagnetic Interference Shielding. Adv. Mater..

[6] Jin X., Wang J., Dai L., Liu X., Li L., Yang Y., Cao Y., Wang W., Wu H., Guo S. (2020). Flame-retardant poly(vinyl alcohol)/MXene multilayered films with outstanding electromagnetic interference shielding and thermal conductive performances. Chem. Eng. J..

[7] Liu J., Zhang H.-B., Sun R., Liu Y., Liu Z., Zhou A., Yu Z.-Z. (2017). Hydrophobic, Flexible, and Lightweight MXene Foams for High-Performance Electromagnetic-Interference Shielding. Adv. Mater..

[8] Weng G.-M., Li J., Alhabeb M., Karpovich C., Wang H., Lipton J., Maleski K., Kong J., Shaulsky E., Elimelech M. (2018). Layer-by-Layer Assembly of Cross-Functional Semi-transparent MXene-Carbon Nanotubes Composite Films for Next-Generation Electromagnetic Interference Shielding. Adv. Funct. Mater..

[9] Zhou M., Wang J., Wang G., Zhao Y., Tang J., Pan J., Ji G. (2022). Lotus leaf-inspired and multifunctional Janus carbon felt@Ag composites enabled by in situ asymmetric modification for electromagnetic protection and low-voltage joule heating. Compos. Part B Eng..

[10] Zhang X.-Y., Wang H.-Q., Sun Y., Zhou W., Wang J. (2021). Facile synthesis of aqueous silver nanoparticles and silver/molybdenum disulfide nanocomposites and investigation of their nonlinear optical properties. Tungsten.

[11] Yang Y., Wu N., Li B., Liu W., Pan F., Zeng Z., Liu J. (2022). Biomimetic Porous MXene Sediment-Based Hydrogel for High-Performance and Multifunctional Electromagnetic Interference Shielding. ACS Nano.

[12] Zheng X., Zhang S., Zhou M., Lu H., Guo S., Zhang Y., Li C., Tan S.C. (2023). MXene Functionalized, Highly Breathable and Sensitive Pressure Sensors with Multi-Layered Porous Structure. Adv. Funct. Mater..

[13] Yoon J., Kim S., Park K.H., Lee S., Kim S.J., Lee H., Oh T., Koo C.M. (2023). Biocompatible and Oxidation-Resistant Ti3C2Tx MXene with Halogen-Free Surface Terminations. Small Methods.

[14] Wu N., Yang Y., Wang C., Wu Q., Pan F., Zhang R., Liu J., Zeng Z. (2023). Ultrathin Cellulose Nanofiber Assisted Ambient-Pressure-Dried, Ultralight, Mechanically Robust, Multifunctional MXene Aerogels. Adv. Mater..

[15] Chen F.-H., Xie H.-B., Huo M.-S., Wu H., Li L.-J., Jiang Z.-Y. (2022). Effects of magnetic field and hydrostatic pressure on the antiferromagnetic–ferromagnetic transition and magneto-functional properties in Hf_1-x_Ta_x_Fe_2_ alloys. Tungsten.

[16] Zou G., Zhang Z., Guo J., Liu B., Zhang Q., Fernandez C., Peng Q. (2016). Synthesis of MXene/Ag Composites for Extraordinary Long Cycle Lifetime Lithium Storage at High Rates. ACS Appl. Mater. Interfaces.

[17] Zhu J., Ha E., Zhao G., Zhou Y., Huang D., Yue G., Hu L., Sun N., Wang Y., Lee L.Y.S. (2017). Recent advance in MXenes: A promising 2D material for catalysis, sensor and chemical adsorption. Coord. Chem. Rev..

[18] Wang X., Chen Y., Hu F., Zhang S., Fan B., Min Z., Zhang R., Zhao B., Wang H., Lu H. (2022). Electromagnetic Interference Shielding Performance of Flexible, Hydrophobic Honeycomb-Structured Ag@Ti3C2Tx Composites. Adv. Electron. Mater..

[19] Song D., Jiang X., Li Y., Lu X., Luan S., Wang Y., Li Y., Gao F. (2019). Metal−organic frameworks-derived MnO_2_/Mn_3_O_4_ microcuboids with hierarchically ordered nanosheets and Ti_3_C_2_ MXene/Au NPs composites for electrochemical pesticide detection. J. Hazard. Mater..

[20] Zeng Z., Jin H., Chen M., Li W., Zhou L., Zhang Z. (2016). Lightweight and Anisotropic Porous MWCNT/WPU Composites for Ultrahigh Performance Electromagnetic Interference Shielding. Adv. Funct. Mater..

[21] Nguyen V.-T., Min B.K., Yi Y., Kim S.J., Choi C.-G. (2020). MXene(Ti3C2TX)/graphene/PDMS composites for multifunctional broadband electromagnetic interference shielding skins. Chem. Eng. J..

[22] Soomro R.A., Zhang P., Fan B., Wei Y., Xu B. (2023). Progression in the Oxidation Stability of MXenes. Nano-Micro Lett..

[23] Bera R., Maitra A., Paria S., Karan S.K., Das A.K., Bera A., Si S.K., Halder L., De A., Khatua B.B. (2018). An approach to widen the electromagnetic shielding efficiency in PDMS/ferrous ferric oxide decorated RGO–SWCNH composite through pressure induced tunability. Chem. Eng. J..

[24] Wang L., Zhang M., Yang B., Tan J., Ding X. (2020). Highly Compressible, Thermally Stable, Light-Weight, and Robust Aramid Nanofibers/Ti3AlC2 MXene Composite Aerogel for Sensitive Pressure Sensor. ACS Nano.

[25] Zhang B., Wang W., Tian M., Ning N., Zhang L. (2020). Preparation of aramid nanofiber and its application in polymer reinforcement: A review. Eur. Polym. J..

[26] Ma Z., Kang S., Ma J., Shao L., Wei A., Liang C., Gu J., Yang B., Dong D., Wei L. (2019). High-Performance and Rapid-Response Electrical Heaters Based on Ultraflexible, Heat-Resistant, and Mechanically Strong Aramid Nanofiber/Ag Nanowire Nanocomposite Papers. ACS Nano.

[27] Chen S., Wang Y., Fei B., Long H., Wang T., Zhang T., Chen L. (2022). Development of a flexible and highly sensitive pressure sensor based on an aramid nanofiber-reinforced bacterial cellulose nanocomposite membrane. Chem. Eng. J..

[28] Yan Z., Ding Y., Huang M., Li J., Han Q., Yang M., Li W. (2023). MXene/CNTs/Aramid Aerogels for Electromagnetic Interference Shielding and Joule Heating. ACS Appl. Nano Mater..

[29] Huang F.-W., Yang Q.-C., Jia L.-C., Yan D.-X., Li Z.-M. (2021). Aramid nanofiber assisted preparation of self-standing liquid metal-based films for ultrahigh electromagnetic interference shielding. Chem. Eng. J..

[30] Yao J., Zhang L., Yang F., Jiao Z., Tao X., Yao Z., Zheng Y., Zhou J. (2022). Superhydrophobic Ti3C2Tx MXene/aramid nanofiber films for high-performance electromagnetic interference shielding in thermal environment. Chem. Eng. J..

[31] Yang B., Wang L., Zhang M., Luo J., Lu Z., Ding X. (2020). Fabrication, Applications, and Prospects of Aramid Nanofiber. Adv. Funct. Mater..

[32] Zhao S., Zhang H.-B., Luo J.-Q., Wang Q.-W., Xu B., Hong S., Yu Z.-Z. (2018). Highly Electrically Conductive Three-Dimensional Ti3C2Tx MXene/Reduced Graphene Oxide Hybrid Aerogels with Excellent Electromagnetic Interference Shielding Performances. ACS Nano.

[33] Zhao F., Yao Y., Jiang C., Shao Y., Barceló D., Ying Y., Ping J. (2020). Self-reduction bimetallic nanoparticles on ultrathin MXene nanosheets as functional platform for pesticide sensing. J. Hazard. Mater..

[34] Zhang Q., Zhang Z., Zhao D., Wang L., Li H., Zhang F., Huo Y., Li H. (2023). Synergistic photocatalytic-photothermal contribution enhanced by recovered Ag+ ions on MXene membrane for organic pollutant removal. Appl. Catal. B Environ..

[35] Li F., Liu Y.-l., Wang G.-G., Zhang S.-Y., Zhao D.-Q., Fang K., Zhang H.-Y., Yang H.Y. (2022). 3D porous H-Ti3C2Tx films as free-standing electrodes for zinc ion hybrid capacitors. Chem. Eng. J..

[36] Jiang Y., Zhang X., Pei L., Yue S., Ma L., Zhou L., Huang Z., He Y., Gao J. (2018). Silver nanoparticles modified two-dimensional transition metal carbides as nanocarriers to fabricate acetycholinesterase-based electrochemical biosensor. Chem. Eng. J..

[37] Li K., Jiao T., Xing R., Zou G., Zhou J., Zhang L., Peng Q. (2018). Fabrication of tunable hierarchical MXene@AuNPs nanocomposites constructed by self-reduction reactions with enhanced catalytic performances. Sci. China Mater..

[38] Ma C., Cao W.-T., Zhang W., Ma M.-G., Sun W.-M., Zhang J., Chen F. (2021). Wearable, ultrathin and transparent bacterial celluloses/MXene film with Janus structure and excellent mechanical property for electromagnetic interference shielding. Chem. Eng. J..

[39] Wu X., Hao L., Zhang J., Zhang X., Wang J., Liu J. (2016). Polymer-Ti3C2Tx composite membranes to overcome the trade-off in solvent resistant nanofiltration for alcohol-based system. J. Membr. Sci..

[40] Liu Y., Zhang J., Zhang X., Li Y., Wang J. (2016). Ti3C2Tx Filler Effect on the Proton Conduction Property of Polymer Electrolyte Membrane. ACS Appl. Mater. Interfaces.

[41] Ma Z., Xiang X., Shao L., Zhang Y., Gu J. (2022). Multifunctional Wearable Silver Nanowire Decorated Leather Nanocomposites for Joule Heating, Electromagnetic Interference Shielding and Piezoresistive Sensing. Angew. Chem. Int. Ed..

[42] Wan Y.-J., Zhu P.-L., Yu S.-H., Sun R., Wong C.-P., Liao W.-H. (2018). Anticorrosive, Ultralight, and Flexible Carbon-Wrapped Metallic Nanowire Hybrid Sponges for Highly Efficient Electromagnetic Interference Shielding. Small.

[43] Yuan C., Huang J., Dong Y., Huang X., Lu Y., Li J., Tian T., Liu W., Song W. (2020). Record-High Transparent Electromagnetic Interference Shielding Achieved by Simultaneous Microwave Fabry–Pérot Interference and Optical Antireflection. ACS Appl. Mater. Interfaces.

[44] Zhang X., Wu K., Zhao G., Deng H., Fu Q. (2022). The preparation of high performance Multi-functional porous sponge through a biomimic coating strategy based on polyurethane dendritic colloids. Chem. Eng. J..

[45] Liu H., Huang Z., Chen T., Su X., Liu Y., Fu R. (2022). Construction of 3D MXene/Silver nanowires aerogels reinforced polymer composites for extraordinary electromagnetic interference shielding and thermal conductivity. Chem. Eng. J..

[46] Wang J., Wu X., Wang Y., Zhao W., Zhao Y., Zhou M., Wu Y., Ji G. (2022). Green, Sustainable Architectural Bamboo with High Light Transmission and Excellent Electromagnetic Shielding as a Candidate for Energy-Saving Buildings. Nano-Micro Lett..

[47] Xue T., Yang Y., Yu D., Wali Q., Wang Z., Cao X., Fan W., Liu T. (2023). 3D Printed Integrated Gradient-Conductive MXene/CNT/Polyimide Aerogel Frames for Electromagnetic Interference Shielding with Ultra-Low Reflection. Nano-Micro Lett..

[48] Li M., Chen D., Deng X., Xu B., Li M., Liang H., Wang M., Song G., Zhang T., Liu Y. (2023). Graded Mxene-Doped Liquid Metal as Adhesion Interface Aiming for Conductivity Enhancement of Hybrid Rigid-Soft Interconnection. ACS Appl. Mater. Interfaces.

[49] Gao Y.-N., Wang Y., Yue T.-N., Wang M. (2023). Achieving absorption-type electromagnetic shielding performance in silver micro-tubes/barium Ferrites/Poly(lactic acid) composites via enhancing impedance matching and electric-magnetic synergism. Compos. Part B Eng..

[50] Gao D., Guo S., Zhou Y., Lyu B., Ma J., Zhao P., Pan D., Chen S. (2022). Hydrophobic, flexible electromagnetic interference shielding films derived from hydrolysate of waste leather scraps. J. Colloid Interface Sci..

[51] Cheng R., Wang B., Zeng J., Li J., Xu J., Gao W., Chen K. (2023). Janus-inspired flexible cellulose nanofiber-assisted MXene/Silver nanowire papers with fascinating mechanical properties for efficient electromagnetic interference shielding. Carbon.

[52] Zhou M., Wang J., Tan S., Ji G. (2023). Top-down construction strategy toward sustainable cellulose composite paper with tunable electromagnetic interference shielding. Mater. Today Phys..

[53] Guo T., Zhou D., Deng S., Jafarpour M., Avaro J., Neels A., Heier J., Zhang C. (2023). Rational Design of Ti3C2Tx MXene Inks for Conductive, Transparent Films. ACS Nano.

[54] Jia Y., Pan Y., Wang C., Liu C., Shen C., Pan C., Guo Z., Liu X. (2021). Flexible Ag Microparticle/MXene-Based Film for Energy Harvesting. Nano-Micro Lett..

[55] Luo J., Huo L., Wang L., Huang X., Li J., Guo Z., Gao Q., Hu M., Xue H., Gao J. (2020). Superhydrophobic and multi-responsive fabric composite with excellent electro-photo-thermal effect and electromagnetic interference shielding performance. Chem. Eng. J..

[56] Liu P., Li Y., Xu Y., Bao L., Wang L., Pan J., Zhang Z., Sun X., Peng H. (2018). Stretchable and Energy-Efficient Heating Carbon Nanotube Fiber by Designing a Hierarchically Helical Structure. Small.

[57] Liang L., Li Q., Yan X., Feng Y., Wang Y., Zhang H.-B., Zhou X., Liu C., Shen C., Xie X. (2021). Multifunctional Magnetic Ti3C2Tx MXene/Graphene Aerogel with Superior Electromagnetic Wave Absorption Performance. ACS Nano.

[58] Zhang Y., Li L., Cao Y., Yang Y., Wang W., Wang J. (2023). High-strength, low infrared-emission nonmetallic films for highly efficient Joule/solar heating, electromagnetic interference shielding and thermal camouflage. Mater. Horiz..

[59] Ma J., Wang K., Zhan M. (2015). A comparative study of structure and electromagnetic interference shielding performance for silver nanostructure hybrid polyimide foams. RSC Adv..

[60] Shahzad F., Alhabeb M., Hatter C.B., Anasori B., Man Hong S., Koo C.M., Gogotsi Y. (2016). Electromagnetic interference shielding with 2D transition metal carbides (MXenes). Science.

[61] Shui X., Chung D.D.L. (1997). Nickel filament polymer-matrix composites with low surface impedance and high electromagnetic interference shielding effectiveness. J. Electron. Mater..

[62] Song W.-L., Guan X.-T., Fan L.-Z., Cao W.-Q., Wang C.-Y., Zhao Q.-L., Cao M.-S. (2015). Magnetic and conductive graphene papers toward thin layers of effective electromagnetic shielding. J. Mater. Chem. A.

[63] Ji K., Zhao H., Zhang J., Chen J., Dai Z. (2014). Fabrication and electromagnetic interference shielding performance of open-cell foam of a Cu–Ni alloy integrated with CNTs. Appl Surf Sci..

[64] Ji K., Zhao H., Huang Z., Dai Z. (2014). Performance of open-cell foam of Cu–Ni alloy integrated with graphene as a shield against electromagnetic interference. Mater. Lett..

[65] Kamal Halder K., Sonker R.K., Sachdev V.K., Tomar M., Gupta V. (2018). Study of electrical, dielectric and EMI shielding behavior of copper metal, copper ferrite and PVDF composite. Integr. Ferroelectr..

[66] Zeng Z., Chen M., Pei Y., Seyed Shahabadi S.I., Che B., Wang P., Lu X. (2017). Ultralight and Flexible Polyurethane/Silver Nanowire Nanocomposites with Unidirectional Pores for Highly Effective Electromagnetic Shielding. ACS Appl. Mater. Interfaces.

[67] Yu Y.-H., Ma C.-C.M., Teng C.-C., Huang Y.-L., Lee S.-H., Wang I., Wei M.-H. (2012). Electrical, morphological, and electromagnetic interference shielding properties of silver nanowires and nanoparticles conductive composites. Mater. Chem. Phys..

[68] Wu S., Zou M., Li Z., Chen D., Zhang H., Yuan Y., Pei Y., Cao A. (2018). Robust and Stable Cu Nanowire@Graphene Core–Shell Aerogels for Ultraeffective Electromagnetic Interference Shielding. Small.

[69] Yan D.-X., Pang H., Li B., Vajtai R., Xu L., Ren P.-G., Wang J.-H., Li Z.-M. (2015). Structured Reduced Graphene Oxide/Polymer Composites for Ultra-Efficient Electromagnetic Interference Shielding. Adv. Funct. Mater..

[70] Yan D.-X., Ren P.-G., Pang H., Fu Q., Yang M.-B., Li Z.-M. (2012). Efficient electromagnetic interference shielding of lightweight graphene/polystyrene composite. J. Mater. Chem..

[71] Xu Y., Wang Q., Cao Y., Wei X., Huang B. (2017). Preparation of a reduced graphene oxide/SiO_2_/Fe_3_O_4_ UV-curing material and its excellent microwave absorption properties. RSC Adv..

[72] Agnihotri N., Chakrabarti K., De A. (2015). Highly efficient electromagnetic interference shielding using graphite nanoplatelet/poly(3,4-ethylenedioxythiophene)–poly(styrenesulfonate) composites with enhanced thermal conductivity. RSC Adv..

[73] Kong L., Yin X., Yuan X., Zhang Y., Liu X., Cheng L., Zhang L. (2014). Electromagnetic wave absorption properties of graphene modified with carbon nanotube/poly(dimethyl siloxane) composites. Carbon.

[74] Chen Z., Xu C., Ma C., Ren W., Cheng H.-M. (2013). Lightweight and Flexible Graphene Foam Composites for High-Performance Electromagnetic Interference Shielding. Adv. Mater..

[75] Xia X., Wang Y., Zhong Z., Weng G.J. (2016). A theory of electrical conductivity, dielectric constant, and electromagnetic interference shielding for lightweight graphene composite foams. J. Appl. Phys..

[76] Shen B., Li Y., Yi D., Zhai W., Wei X., Zheng W. (2016). Microcellular graphene foam for improved broadband electromagnetic interference shielding. Carbon.

[77] Pande S., Chaudhary A., Patel D., Singh B.P., Mathur R.B. (2014). Mechanical and electrical properties of multiwall carbon nanotube/polycarbonate composites for electrostatic discharge and electromagnetic interference shielding applications. RSC Adv..

[78] Al-Saleh M.H., Saadeh W.H., Sundararaj U. (2013). EMI shielding effectiveness of carbon based nanostructured polymeric materials: A comparative study. Carbon.

[79] Arjmand M., Apperley T., Okoniewski M., Sundararaj U. (2012). Comparative study of electromagnetic interference shielding properties of injection molded versus compression molded multi-walled carbon nanotube/polystyrene composites. Carbon.

[80] Yang Y., Gupta M.C., Dudley K.L., Lawrence R.W. (2005). Novel Carbon Nanotube−Polystyrene Foam Composites for Electromagnetic Interference Shielding. Nano Lett..

[81] Huang Y., Li N., Ma Y., Du F., Li F., He X., Lin X., Gao H., Chen Y. (2007). The influence of single-walled carbon nanotube structure on the electromagnetic interference shielding efficiency of its epoxy composites. Carbon.

[82] Sambyal P., Iqbal A., Hong J., Kim H., Kim M.-K., Hong S.M., Han M.-K., Gogotsi Y., Koo C.-M. (2019). Ultralight and Mechanically Robust Ti_3_C_2_T_x_ Hybrid Aerogel Reinforced by Carbon Nanotubes for Electromagnetic Interference Shielding. ACS Appl. Mater. Interfaces.

[83] Crespo M., González M., Elías A.L., Pulickal Rajukumar L., Baselga J., Terrones M., Pozuelo J. (2014). Ultra-light carbon nanotube sponge as an efficient electromagnetic shielding material in the GHz range. Phys. Status Solidi (RRL)–Rapid Res. Lett..

[84] Kuang T., Chang L., Chen F., Sheng Y., Fu D., Peng X. (2016). Facile preparation of lightweight high-strength biodegradable polymer/multi-walled carbon nanotubes nanocomposite foams for electromagnetic interference shielding. Carbon.

[85] Zeng Z., Chen M., Jin H., Li W., Xue X., Zhou L., Pei Y., Zhang H., Zhang Z. (2016). Thin and flexible multi-walled carbon nanotube/waterborne polyurethane composites with high-performance electromagnetic interference shielding. Carbon.

[86] Sheng A., Ren W., Yang Y., Yan D.-X., Duan H., Zhao G., Liu Y.-Q., Li Z.-M. (2020). Multilayer WPU conductive composites with controllable electro-magnetic gradient for absorption-dominated electromagnetic interference shielding. Compos. Part A Appl. Sci. Manuf..

[87] Moglie F., Micheli D., Laurenzi S., Marchetti M., Mariani Primiani V. (2012). Electromagnetic shielding performance of carbon foams. Carbon.

[88] Zhang L., Liu M., Roy S., Chu E.K., See K.Y., Hu X. (2016). Phthalonitrile-Based Carbon Foam with High Specific Mechanical Strength and Superior Electromagnetic Interference Shielding Performance. ACS Appl. Mater. Interfaces.

[89] Moulart A., Marrett C., Colton J. (2004). Polymeric composites for use in electronic and microwave devices. Polym. Eng. Sci..

[90] Ghosh P., Chakrabarti A. (2000). Conducting carbon black filled EPDM vulcanizates: Assessment of dependence of physical and mechanical properties and conducting character on variation of filler loading. Eur. Polym. J..

[91] Bera R., Maiti S., Khatua B.B. (2015). High electromagnetic interference shielding with high electrical conductivity through selective dispersion of multiwall carbon nanotube in poly (ε-caprolactone)/MWCNT composites. J. Appl. Polym. Sci..

[92] Cao W.-T., Chen F.-F., Zhu Y.-J., Zhang Y.-G., Jiang Y.-Y., Ma M.-G., Chen F. (2018). Binary Strengthening and Toughening of MXene/Cellulose Nanofiber Composite Paper with Nacre-Inspired Structure and Superior Electromagnetic Interference Shielding Properties. ACS Nano.

[93] Zhou B., Zhang Z., Li Y., Han G., Feng Y., Wang B., Zhang D.-B., Ma J.-M., Liu C.-T. (2020). Flexible, Robust, and Multifunctional Electromagnetic Interference Shielding Film with Alternating Cellulose Nanofiber and MXene Layers. ACS Appl. Mater. Interfaces.

[94] Zhou J., Thaiboonrod S., Fang J., Cao S., Miao M., Feng X. (2022). In-situ growth of polypyrrole on aramid nanofibers for electromagnetic interference shielding films with high stability. Nano Res..

