# Supplementary material for: Asymmetric and Flexible Ag-MXene/ANFs Composite Papers for Electromagnetic Shielding and Thermal Management
Source: Nanomaterials (Basel). 2023 Sep 21;13(18):2608. doi: 10.3390/nano13182608 (PMC10536414; doi:10.3390/nano13182608)
Supplement: Supplementary file 1 [file nanomaterials-13-02608-s001.zip › nanomaterials-2602257-supplementary.pdf]

Supporting Information

Asymmetric and Flexible Ag-MXene/ANFs Composite Papers for Electromagnetic Shielding and Thermal Management

Xiaoai Ye[a,1], Xu Zhang[a,1], Xinsheng Zhou[a], Guigen Wang[a,b,*]

[a]*Guangdong Provincial Key Laboratory of Semiconductor Optoelectronic Materials and Intelligent Photonic Systems, School of Materials Science and Engineering, Harbin Institute of Technology (Shenzhen), Shenzhen 518055, P. R. China.*

[b]*National Key Laboratory of Science and Technology on Advanced Composites in Special Environments, Harbin Institute of Technology, Harbin 150080, P. R. China.*

*To whom correspondence should be addressed: wangguigen@hit.edu.cn (G. G. Wang).

[1] These authors contributed equally to the work.

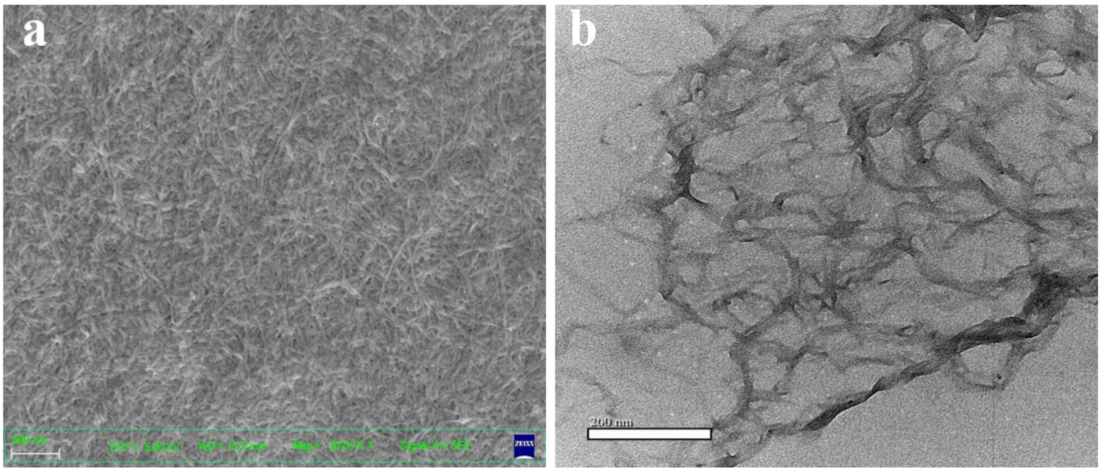

Figure S1. a SEM images of ANFs film. **b** TEM images of ANFs

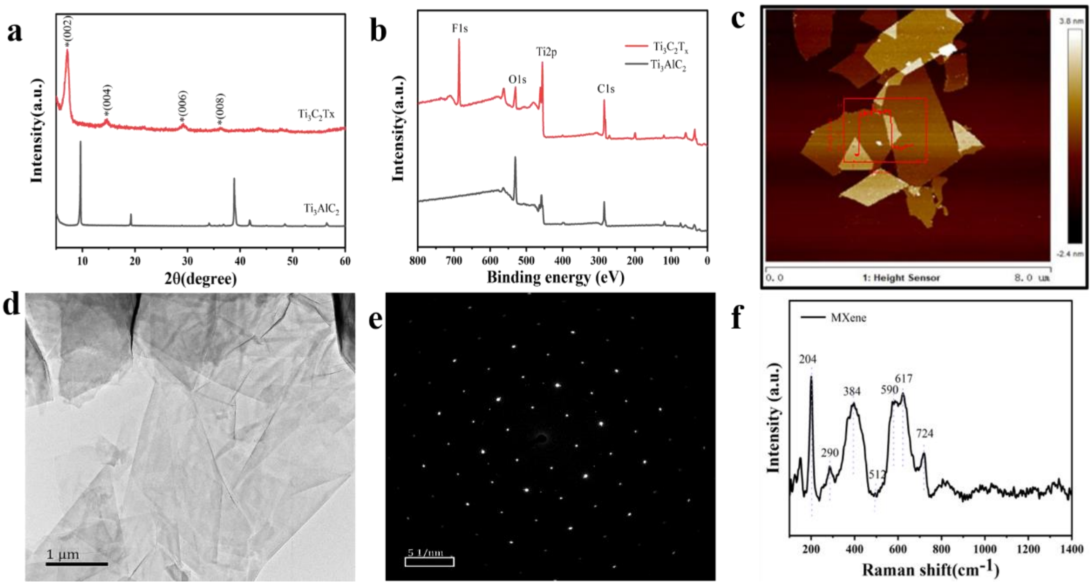

Figure S2. **a** XRD patterns of $Ti_3C_2T_x$ MXene. **b** XPS wide-scan spectra of $Ti_3C_2T_x$ MXene. **c** AFM images of $Ti_3C_2T_x$ MXene. **d** TFM image of $Ti_3C_2T_x$ nanosheets. **e** SAED image of $Ti_3C_2T_x$ nanosheets. **f** Raman spectra of $Ti_3C_2T_x$ nanosheet.

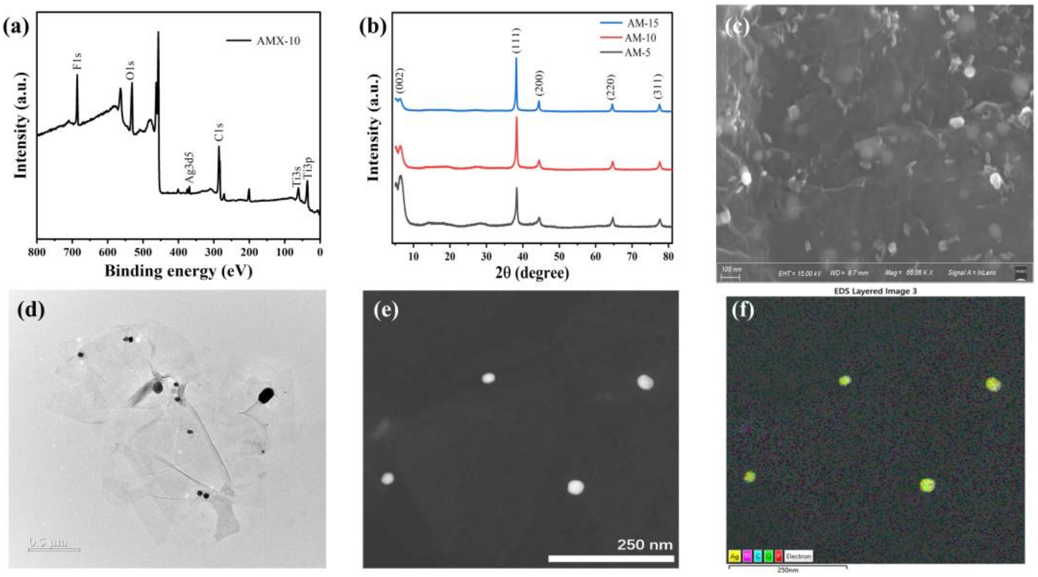

Figure S3. **a** Survey XPS spectra for Ag-MXene hybrids. **b** The XRD patterns of Ag-MXene hybrid nanocomposites. **c** SEM images of Ag-MXene hybrid nanocomposites. **d** TEM images of formed Ag-MXene hybrid nanocomposites. **e-f** EDS spectra of Ag-MXene Hybrids.

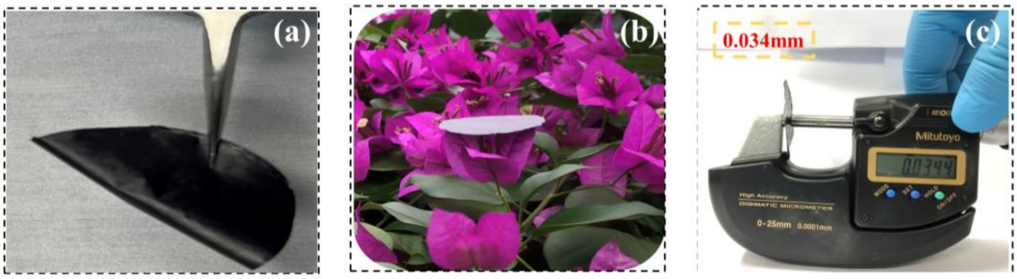

Figure S4. a-c Digital images of the as-obtained AMAGM composite paper.

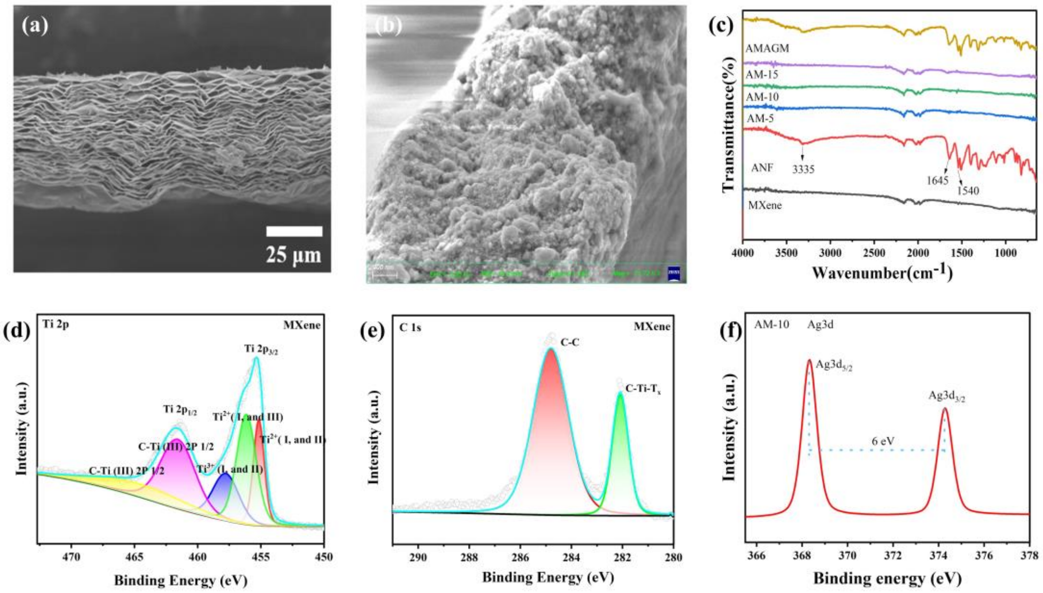

Figure S5. Morphology and structure characterization. **a** Cross-sectional SEM images of $Ti_3C_2T_x$ nanosheets paper. **b** Cross-sectional SEM images of Ag-MXene composite paper. **c** FTIR spectra of MXene, ANFs and AMAGM nanocomposite papers. XPS surveys of delaminated $Ti_3C_2T_x$ nanosheets and Ag-MXene nanocomposites. **d** High-resolution spectra of C 1s for MXene. **e** High-resolution spectra of Ti 2p for MXene. **f** High-resolution XPS spectra of Ag 3d for Ag-MXene hybrids.

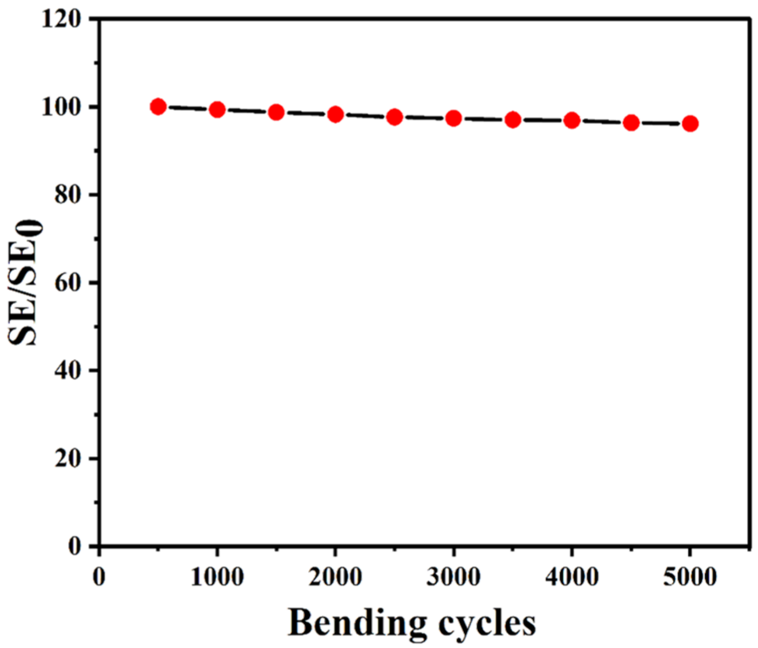

Figure S6. EMI SE variation of the AMAGM composite paper after 5000 cycles of bending deformation.

Table S1. Performance comparison of AMAGM nanocomposite papers with previous reported EMI shielding materials.

Type	Sample	Materials	Thickness (mm)	SE (dB)	SSE/t (dB cm^2g^{-1})	Refs.
Metal-based	1	Ag NW	0.5	35	12416	[1]
	2	Cu foil	0.01	70	7812	[2]
	3	Ni fiber/PES	2.85	58	109	[3]
	4	Ni filaments/PES	2.85	87	165	[4]
	5	Cu-Ni-CNT	1.5	54.6	1580	[5]
	6	Cu-Ni foam	1.5	25	690	[6]
	7	Copper	1.5	90	3200	[7]
	8	Ag NWs/WPU foam	2.3	80	10907	[8]
	9	Ag NW/epoxy	0.4	25.09	5018	[9]
	10	Cu NW@ graphene aerogels	3.46	52.5	3921.8	[10]
rGO-based	11	rGO	2.5	45.1	692	[11]
	12	rGO/PS	2	29	258	[12]
	13	rGO/Fe_3O_4	0.3	24	1033	[13]
	14	rGO/PEDOT	0.8	70	841	[14]
Graphe	15	Graphene	2	75	4165	[15]

ne-based		foam/CNT/PDMS				
	16	Graphene/PDMS foam	1	30	5000	[16]
	17	Graphene/PDMS	0.1	20	3330	[17]
	18	Microcellular graphene foam	0.3	25.2	14000	[18]
CNT-based	19	MWCNT/WPU	0.1	21.1	5410	[19]
	20	MWCNT/PC	2.1	39	164	[20]
	21	MWCNT/ABS	1.1	50	433	[21]
	22	MWCNT/PS	2	30	285	[22]
	23	SWCNT/PS	1.2	18.5	275	[23]
	24	SWCNT/epoxy	2	25	720	[24]
	25	MXCNT	3	104	8253	[25]
	26	CNT sponge	2.38	22	4522	[26]
	27	MWCNT/PLA foam	2.5	23	3080	[27]
	28	MWCNT/WPU	0.32	50	3408	[28]
	29	Fe_3O_4@rGO/MWCNT/WPU	2.3	23	4991	[29]

Carbon -based	30	Carbon foam	0.2	40	1250	[30]
	31	Carbon/PN resin	0.2	51.2	1705	[31]
	32	CB/ABS	1.1	20	1905	[32]
	33	CB/EPDM	2	18	1500	[33]
	34	Commercial carbon foam	2	40	1250	[34]
MXene -based	35	$Ti_3C_2T_x$/CNFs	0.047	24	2647	[35]
	36	$Ti_3C_2T_x$/rGO/epoxy	2	56.4	9400	[36]
	37	CNF@MXene	0.035	39.67029	6800	[37]
Conductive Polymer-based	38	PPy/ANFs	0.076	41.69	2762.5	[38]
This work	39	AMA mixture	0.0344	37.5	6319	
	40	AMAGM	0.0344	62	11923	

Supplementary References

[1] Ma J, Wang K, Zhan M. A comparative study of structure and electromagnetic

interference shielding performance for silver nanostructure hybrid polyimide foams. RSC Advances. 2015;5(80):65283-96.
[2] Shahzad F, Alhabeb M, Hatter CB, Anasori B, Man Hong S, Koo CM, et al. Electromagnetic interference shielding with 2D transition metal carbides (MXenes). Science. 2016;353(6304):1137-40.
[3] Shui X, Chung DDL. Nickel filament polymer-matrix composites with low surface impedance and high electromagnetic interference shielding effectiveness. J Electron Mater. 1997;26(8):928-34.
[4] Song W-L, Guan X-T, Fan L-Z, Cao W-Q, Wang C-Y, Zhao Q-L, et al. Magnetic and conductive graphene papers toward thin layers of effective electromagnetic shielding. Journal of Materials Chemistry A. 2015;3(5):2097-107.
[5] Ji K, Zhao H, Zhang J, Chen J, Dai Z. Fabrication and electromagnetic interference shielding performance of open-cell foam of a Cu–Ni alloy integrated with CNTs. Appl Surf Sci. 2014;311:351-6.
[6] Ji K, Zhao H, Huang Z, Dai Z. Performance of open-cell foam of Cu–Ni alloy integrated with graphene as a shield against electromagnetic interference. Materials Letters. 2014;122:244-7.
[7] Kamal Halder K, Sonker RK, Sachdev VK, Tomar M, Gupta V. Study of electrical, dielectric and EMI shielding behavior of copper metal, copper ferrite and PVDF composite. Integrated Ferroelectrics. 2018;194(1):80-7.
[8] Zeng Z, Chen M, Pei Y, Seyed Shahabadi SI, Che B, Wang P, et al. Ultralight and Flexible Polyurethane/Silver Nanowire Nanocomposites with Unidirectional Pores for Highly Effective Electromagnetic Shielding. ACS Applied Materials & Interfaces. 2017;9(37):32211-9.
[9] Yu Y-H, Ma C-CM, Teng C-C, Huang Y-L, Lee S-H, Wang I, et al. Electrical, morphological, and electromagnetic interference shielding properties of silver nanowires and nanoparticles conductive composites. Materials Chemistry and Physics. 2012;136(2):334-40.
[10] Wu S, Zou M, Li Z, Chen D, Zhang H, Yuan Y, et al. Robust and Stable Cu Nanowire@Graphene Core–Shell Aerogels for Ultraeffective Electromagnetic

Interference Shielding. Small. 2018;14(23):1800634.
[11] Yan D-X, Pang H, Li B, Vajtai R, Xu L, Ren P-G, et al. Structured Reduced Graphene Oxide/Polymer Composites for Ultra-Efficient Electromagnetic Interference Shielding. Advanced Functional Materials. 2015;25(4):559-66.
[12] Yan D-X, Ren P-G, Pang H, Fu Q, Yang M-B, Li Z-M. Efficient electromagnetic interference shielding of lightweight graphene/polystyrene composite. Journal of Materials Chemistry. 2012;22(36):18772-4.
[13] Xu Y, Wang Q, Cao Y, Wei X, Huang B. Preparation of a reduced graphene oxide/SiO_2/Fe_3O_4 UV-curing material and its excellent microwave absorption properties. RSC Advances. 2017;7(29):18172-7.
[14] Agnihotri N, Chakrabarti K, De A. Highly efficient electromagnetic interference shielding using graphite nanoplatelet/poly(3,4-ethylenedioxythiophene)–poly(styrenesulfonate) composites with enhanced thermal conductivity. RSC Advances. 2015;5(54):43765-71.
[15] Kong L, Yin X, Yuan X, Zhang Y, Liu X, Cheng L, et al. Electromagnetic wave absorption properties of graphene modified with carbon nanotube/poly(dimethyl siloxane) composites. Carbon. 2014;73:185-93.
[16] Chen Z, Xu C, Ma C, Ren W, Cheng H-M. Lightweight and Flexible Graphene Foam Composites for High-Performance Electromagnetic Interference Shielding. Advanced Materials. 2013;25(9):1296-300.
[17] Xia X, Wang Y, Zhong Z, Weng GJ. A theory of electrical conductivity, dielectric constant, and electromagnetic interference shielding for lightweight graphene composite foams. Journal of Applied Physics. 2016;120(8).
[18] Shen B, Li Y, Yi D, Zhai W, Wei X, Zheng W. Microcellular graphene foam for improved broadband electromagnetic interference shielding. Carbon. 2016;102:154-60.
[19] Zeng Z, Jin H, Chen M, Li W, Zhou L, Zhang Z. Lightweight and Anisotropic Porous MWCNT/WPU Composites for Ultrahigh Performance Electromagnetic Interference Shielding. Advanced Functional Materials. 2016;26(2):303-10.
[20] Pande S, Chaudhary A, Patel D, Singh BP, Mathur RB. Mechanical and electrical properties of multiwall carbon nanotube/polycarbonate composites for electrostatic

discharge and electromagnetic interference shielding applications. RSC Advances. 2014;4(27):13839-49.

[21] Al-Saleh MH, Saadeh WH, Sundararaj U. EMI shielding effectiveness of carbon based nanostructured polymeric materials: A comparative study. Carbon. 2013;60:146-56.

[22] Arjmand M, Apperley T, Okoniewski M, Sundararaj U. Comparative study of electromagnetic interference shielding properties of injection molded versus compression molded multi-walled carbon nanotube/polystyrene composites. Carbon. 2012;50(14):5126-34.

[23] Yang Y, Gupta MC, Dudley KL, Lawrence RW. Novel Carbon Nanotube−Polystyrene Foam Composites for Electromagnetic Interference Shielding. Nano Letters. 2005;5(11):2131-4.

[24] Huang Y, Li N, Ma Y, Du F, Li F, He X, et al. The influence of single-walled carbon nanotube structure on the electromagnetic interference shielding efficiency of its epoxy composites. Carbon. 2007;45(8):1614-21.

[25] Sambyal P, Iqbal A, Hong J, Kim H, Kim M-K, Hong SM, et al. Ultralight and Mechanically Robust $Ti_3C_2T_x$ Hybrid Aerogel Reinforced by Carbon Nanotubes for Electromagnetic Interference Shielding. ACS Applied Materials & Interfaces. 2019;11(41):38046-54.

[26] Crespo M, González M, Elías AL, Pulickal Rajukumar L, Baselga J, Terrones M, et al. Ultra-light carbon nanotube sponge as an efficient electromagnetic shielding material in the GHz range. physica status solidi (RRL) – Rapid Research Letters. 2014;8(8):698-704.

[27] Kuang T, Chang L, Chen F, Sheng Y, Fu D, Peng X. Facile preparation of lightweight high-strength biodegradable polymer/multi-walled carbon nanotubes nanocomposite foams for electromagnetic interference shielding. Carbon. 2016;105:305-13.

[28] Zeng Z, Chen M, Jin H, Li W, Xue X, Zhou L, et al. Thin and flexible multi-walled carbon nanotube/waterborne polyurethane composites with high-performance

electromagnetic interference shielding. Carbon. 2016;96:768-77.
[29] Sheng A, Ren W, Yang Y, Yan D-X, Duan H, Zhao G, et al. Multilayer WPU conductive composites with controllable electro-magnetic gradient for absorption-dominated electromagnetic interference shielding. Composites Part A: Applied Science and Manufacturing. 2020;129:105692.
[30] Moglie F, Micheli D, Laurenzi S, Marchetti M, Mariani Primiani V. Electromagnetic shielding performance of carbon foams. Carbon. 2012;50(5):1972-80.
[31] Zhang L, Liu M, Roy S, Chu EK, See KY, Hu X. Phthalonitrile-Based Carbon Foam with High Specific Mechanical Strength and Superior Electromagnetic Interference Shielding Performance. ACS applied materials & interfaces. 2016;8 11:7422-30.
[32] Moulart A, Marrett C, Colton J. Polymeric composites for use in electronic and microwave devices. Polymer Engineering & Science. 2004;44(3):588-97.
[33] Ghosh P, Chakrabarti A. Conducting carbon black filled EPDM vulcanizates: assessment of dependence of physical and mechanical properties and conducting character on variation of filler loading. European Polymer Journal. 2000;36(5):1043-54.
[34] Bera R, Maiti S, Khatua BB. High electromagnetic interference shielding with high electrical conductivity through selective dispersion of multiwall carbon nanotube in poly (ε-caprolactone)/MWCNT composites. Journal of Applied Polymer Science. 2015;132(26).
[35] Cao W-T, Chen F-F, Zhu Y-J, Zhang Y-G, Jiang Y-Y, Ma M-G, et al. Binary Strengthening and Toughening of MXene/Cellulose Nanofiber Composite Paper with Nacre-Inspired Structure and Superior Electromagnetic Interference Shielding Properties. ACS Nano. 2018;12(5):4583-93.
[36] Zhao S, Zhang H-B, Luo J-Q, Wang Q-W, Xu B, Hong S, et al. Highly Electrically Conductive Three-Dimensional Ti3C2Tx MXene/Reduced Graphene Oxide Hybrid Aerogels with Excellent Electromagnetic Interference Shielding Performances. ACS Nano. 2018;12(11):11193-202.
[37] Zhou B, Zhang Z, Li Y, Han G, Feng Y, Wang B, et al. Flexible, Robust, and

Multifunctional Electromagnetic Interference Shielding Film with Alternating Cellulose Nanofiber and MXene Layers. ACS Applied Materials & Interfaces. 2020;12(4):4895-905.
[38] Zhou J, Thaiboonrod S, Fang J, Cao S, Miao M, Feng X. In-situ growth of polypyrrole on aramid nanofibers for electromagnetic interference shielding films with high stability. Nano Research. 2022;15(9):8536-45.
